# Structural determinants of PINK1 topology and dual subcellular distribution

**DOI:** 10.1186/1471-2121-11-90

**Published:** 2010-11-22

**Authors:** William Lin, Un Jung Kang

**Affiliations:** 1 Department of Neurology, University of Chicago Medical Center, Chicago, Illinois 60637, USA

## Abstract

**Background:**

PINK1 is a mitochondria-targeted kinase that constitutively localizes to both the mitochondria and the cytosol. The mechanism of how PINK1 achieves cytosolic localization following mitochondrial processing remains unknown. Understanding PINK1 subcellular localization will give us insights into PINK1 functions and how mutations in PINK1 lead to Parkinson's disease. We asked how the mitochondrial localization signal, the transmembrane domain, and the kinase domain participate in PINK1 localization.

**Results:**

We confirmed that PINK1 mitochondrial targeting signal is responsible for mitochondrial localization. Once inside the mitochondria, we found that both PINK1 transmembrane and kinase domain are important for membrane tethering and cytosolic-facing topology. We also showed that PINK1 dual subcellular distribution requires both Hsp90 interaction with the kinase domain and the proteolysis at a cleavage site downstream of the transmembrane domain because removal of this cleavage site completely abolished cytosolic PINK1. In addition, the disruption of the Hsp90-PINK1 interaction increased mitochondrial PINK1 level.

**Conclusion:**

Together, we believe that once PINK1 enters the mitochondria, PINK1 adopts a tethered topology because the transmembrane domain and the kinase domain prevent PINK1 forward movement into the mitochondria. Subsequent proteolysis downstream of the transmembrane domain then releases PINK1 for retrograde movement while PINK1 kinase domain interacts with Hsp90 chaperone. The significance of this dual localization could mean that PINK1 has compartmental-specific functions.

## Background

Nuclear-encoded mitochondrial proteins synthesized in the cytosol are targeted to the mitochondria by one of two types of targeting signals, a hydrophobic presequence (MLS) and/or a cryptic internal sequence [1]. The MLS directs the precursor protein to the translocase of the outer membrane (TOMM) where translocation begins. In addition, the MLS affects the precursor import efficiency as determined by the length of signal peptide [2] and encodes the submitochondrial localization of mitochondrial proteins after mitochondrial processing, as exemplified by the presence of a cleavable or non-cleavable stop-transfer signal [3]. Redistribution after mitochondrial processing can also be affected by protein folding, even though most precursor translocation requires unfolding. Of the two reported examples of protein folding affecting mitochondrial import, the propeller domain of PP2A/Bβ2 subunit arrests the import process and becomes on OMM protein [4] whereas rapid folding of yeast fumarase during the import favors the retrograde movement for a cytosolic localization [5]. Interestingly, there are only a handful of proteins that distribute between the mitochondria and cytosol in a constitutive manner, fumarase being the most studied example. It has been demonstrated that fumarase has a 30%/70% mitochondria/cytosol isoprotein distribution and this dual localization occurs after mitochondrial processing [6].

The *PINK1 *gene encodes a kinase protein that contains an N-terminal MLS and mutations in *PINK1 *are linked to a recessive form of Parkinson's disease. Using a heterologous expression system, varying lengths of PINK1 MLS were tested (1-33aa, 1-77aa, and 1-156aa) and all PINK1 MLS-GFP fusion proteins co-localized with mitochondrial markers, such as mitotracker or TOM20 fluorescence [7-9]. These studies proved that PINK1 MLS is sufficient for mitochondrial targeting. The submitochondrial localization of PINK1, by biochemical fractionation, shows that all forms of PINK1 are found at the outer membrane, intermembrane space, and inner membrane, but not the matrix [8,10]. However, the subcellular localization of endogenous and overexpressed PINK1 in cell culture models show that PINK1 does not solely localize to the mitochondrial fraction, as cytosolic and microsomal fractions are found to contain all cleaved forms of PINK1 [7,11-13]. Overexpression of cytosolic PINK1, one that lacks the MLS, exhibits protective function against MPTP toxicity in mice and in cell culture [14]. Also, proteins found to associate with PINK1 are either cytosolic (Parkin, DJ-1, Hsp90, and Cdc37 [12,13,15,16]) or cytosolically exposed (Miro and Milton [17]). Only HtrA2 and TRAP1 are found to associate with PINK1 in the mitochondria [10,18]. Currently no studies have examined the function of the mitochondrial form of PINK1 in the absence of the cytosolic PINK1.

Several important questions arise from PINK1 dual localization: what purpose does the PINK1 MLS serve if a functional PINK1 protein is also found in the cytosol? How does PINK1 redistribute after mitochondrial processing? Is the function of PINK1 different in mitochondria as compared to the cytosol? We are very interested to understand the mechanism behind PINK1 dual distribution, especially given the evidence that the mitochondrial pool of PINK1 is tethered to the OMM (with the kinase domain exposed to the cytosol) and removal of the PINK1 transmembrane domain mislocalizes PINK1 inside the mitochondria [19]. We previously showed that PINK1 cleaved forms are generated from the mitochondrial processing of PINK1 precursor, thus suggesting that PINK1 cytosolic redistribution occurs after cleavage [12]. We hypothesize that while the PINK1 MLS can direct proteins to the mitochondria, the required interaction between the PINK1 kinase domain and Hsp90 chaperone favors a retrograde movement, thus resulting in a cytosolic localization. To test our hypothesis, we fused wildtype PINK1 as well as PINK1 mutant that lacks Hsp90 chaperone interaction with other known MLS and examined the cytosolic and mitochondrial distribution of these proteins when expressed in a cell culture model.

## Results

### PINK1 N-terminal cleavages occur before and after PINK1 transmembrane domain

**Figure 1:**
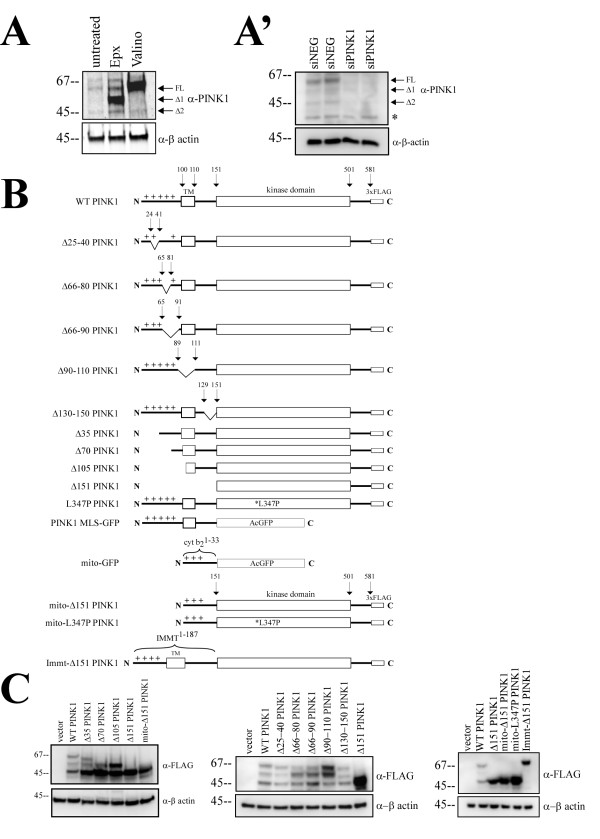
**Design and expression of various PINK1 constructs**. A) Three endogenous PINK1 forms can be detected and identified in Hela cells using the Odyssey Infrared Imaging. Valinomycin (Δψm dissipater) increases PINK1 FL, epoxomicin (proteasome inhibitor) increases both PINK1 Δ1 and Δ2. A') PINK1 siRNA knocked down all three endogenous PINK1 forms. Asterisk denotes non-specific band from α-PINK1 antibodies. B) Schematic diagram of various PINK1 deletion mutants and various MLS-PINK1 fusion constructs. Cyt b2 is yeast cytochrome b2 MLS from 1-33 amino acids. IMMT is human mitofilin MLS from 1-187 amino acids. C) Protein expression of aforementioned PINK1 constructs relative to WT PINK1 is compared by anti-FLAG western blot. All PINK1 deletion mutants express PINK1 Δ2 form, corresponding to Δ151 PINK1 molecular weight. Immt-Δ151 PINK1 is detected as a single protein product.

At first glance, PINK1 MLS is similar either to those of inner membrane (uncleaved transmembrane anchor) or intermembrane space proteins (bipartite presequence). The difference between these two signals is the cleavage site after the transmembrane domain, which would determine whether or not the protein is anchored. Overexpression of WT PINK1 in cell lines leads to the generation of three or more PINK1 forms, suggesting the presence of multiple cleavage sites [7,9,11,12]. The pattern of endogenous PINK1 protein is debatable due to low endogenous PINK1 expression and the lack of a high affinity antibody although it is generally accepted and agreed upon that at least two endogenous PINK1 bands are detectable-the full length and a cleaved form around 55 kDa [13,20,21]. A most recent paper showed three endogenous bands [21]. We and others have previously demonstrated that endogenous PINK1 behaves similarly to the overexpressed PINK1 counterparts in that PINK1 FL accumulates under valinomycin treatment and PINK1 Δ1 and Δ2 accumulate under proteasome inhibitor treatment [9,12,22]. Using these two chemical inhibitors, we first wanted to establish that Hela cells express three forms of endogenous PINK1. We observed that valinomycin treatment led to the increase of PINK1 FL, and epoxomicin treatment increased two lower protein bands when compared to untreated cells (Figure 1A). With epoxomicin, the heavily accumulated protein is PINK1 Δ1 and the protein around 45 kDa is the PINK1 Δ2 form. We also tested the specificity of these three PINK1 bands by using siRNA to knockdown endogenous PINK1. In two independent siPINK1 transfections, western blot showed all three endogenous PINK1 proteins were decreased (Figure 1A'), confirming the hypothesis that endogenous PINK1 also expresses two cleaved forms. In addition, we do not believe that the PINK1 Δ2 form is a mere degradation product because our previous metabolic labeling data showed that PINK1 Δ2 form is most stable protein of all PINK1 forms [12].

Potential mitochondrial processing motifs have been examined for PINK1 MLS, where one predicted site is mapped at amino acid 35 and the second site around amino acids 77 [8]. Both predicted cleavage sites correspond with the consensus R-2 or R-10 matrix processing motif [23]. The second processing consensus motif is upstream of the PINK1 transmembrane domain (predicted from amino acids 90-110) and proteolysis at this site can generate a protein with similar molecular weight to PINK1 Δ1 form. We were first interested in determining the approximate molecular sizes of each PINK1 cleaved products, which might yield clues about possible proteolytic sites. We constructed and expressed N-terminal serial truncation mutants, Δ35 PINK1, Δ70 PINK1, Δ105 PINK1, and Δ151 PINK1. By western blot, Δ70 and Δ105 PINK1 showed proteins expressed as similar molecular weight as WT PINK1 Δ1 and Δ2 cleaved products (Figure 1C). We also observed that Δ151 PINK1 was only expressed as a single form, corresponding to the smallest band in all of the PINK1 constructs (Figure 1C). Data from these truncation mutants suggests that possible cleavage sites are within aa70-105 and aa105-151. This is similar to a recent publication using serial N-terminal deletion PINK1 constructs which suggested that the first cleavage site resides between aa91-101 [19], placing the putative cleavage site within the transmembrane domain. Since the disruption of N-terminal sequences may have affected mitochondrial targeting and cleavage, we also studied internal deletion mutants to map out the proteolytic sites in the PINK1 MLS (Figure 1B). By targeting the predicted cleavage sites in the PINK1 N-terminus, we truncated from aa25-40, aa66-80, aa66-90, aa90-110, and aa130-150. Unfortunately none of the internal deletions were able to abolish PINK1 cleavage (Figure 1C), illustrating the complexity of PINK1 MLS proteolysis. We did find that Δ25-40 PINK1 was consistent with Δ35 PINK1 in ruling out the cleavage site predicted at position 35. Based on N-terminal deletion mutants we predicted that a second cleavage site resides downstream of the transmembrane domain.

### PINK1 transmembrane and kinase domain determine PINK1 subcellular distribution

**Figure 2:**
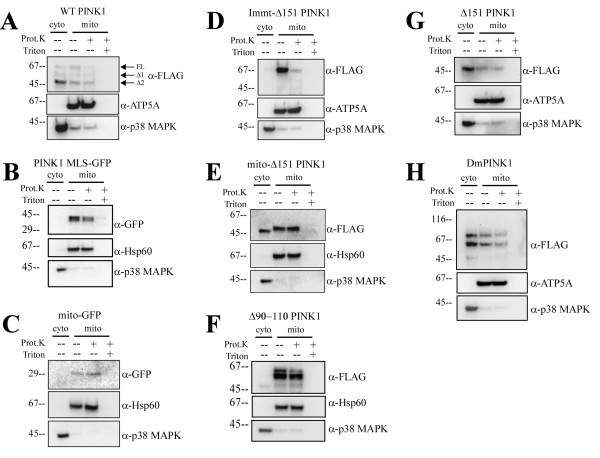
**Subcellular distribution of various PINK1 mutants in Hela cells by fractionation**. A) PINK1 MLS-GFP localizes to the mitochondria and is partially sensitive to proteinase K digestion. B) mito-GFP localizes to the mitochondria and is not digested by proteinase K. C) Overexpression of wildtype PINK1-flag displays dual localization for all forms of PINK1. D) Immt-Δ151 PINK1 localizes to the mitochondria and is sensitive to proteinase K digestion. E) mito-Δ151 PINK1 localizes equally in both cytosol and mitochondria, where the mitochondria fraction is not digested by proteinase K. F) Δ90-110 PINK1 mostly localizes to mitochondrial fraction with a small portion of cleaved form redistributed to the cytosolic fraction. G) Δ151 PINK1 localizes mainly in the cytosol with a small pool in the mitochondria. H) Drosophila PINK1-flag (DmPINK1) transfected in Hela cells also distributed to both cytosolic and mitochondrial fractions, similar to human PINK1. ATP5A, the α-subunit of ATP synthase, marks the mitochondria. p38 MAPK marks the cytosol.

As demonstrated before, WT PINK1 overexpression showed dual subcellular distribution with all three forms found in both mitochondrial and cytosolic fractions (Figure 2A). We asked how elements in the PINK1 structure can contribute to the mechanism behind PINK1 dual distribution. PINK1 protein contains three easily identifiable elements, an N-terminal MLS, a TM, and a C-terminal kinase domain. In general, the presence of a transmembrane domain in the MLS serves as a stop-transfer, or sorting signal, that prevents mitochondrial proteins from matrix import. *We tested three most feasable hypotheses: 1) PINK1 TM serves as a stop-transfer signal, given that PINK1 is not found in the matrix and PINK1 mislocalized to the matrix compartment when the TM was deleted *[19], *2) the cleavage after the transmembrane domain allows mitochondrial pool of PINK1 to become soluble, thus making it possible to redistribute to the cytosol, 3) the kinase domain interaction with Hsp90 in the cytosol prevents PINK1 from complete mitochondrial import, thus PINK1 adopts a topology where the kinase domain is exposed to the cytosolic face on the OMM.*

**Figure 3:**
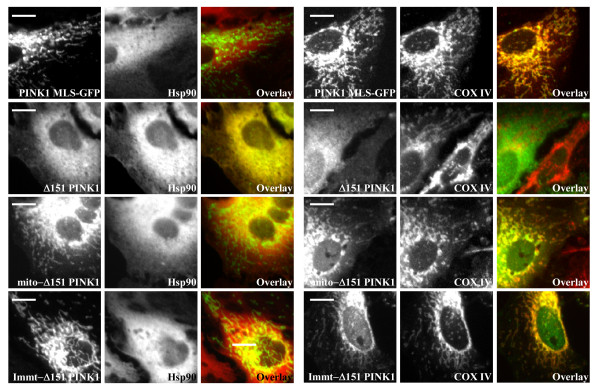
**Immunofluorescence of PINK1-MLS GFP, Δ151 PINK1, mito-Δ151 PINK1, and Immt-Δ151 PINK1 in Hela cells**. Cells transiently transfected with aforementioned constructs are also co-stained with either cytosol marker Hsp90 or mitochondria marker COX IV. Confocal images are analyzed with ImageJ and the yellow color in the overlay panels represents co-localization. Scale bar = 5 μm.

We first tested the involvement of the TM in topology and dual distribution by using PINK1 MLS-GFP, where the PINK1 TM is intact but the C-terminal kinase domain is now replaced with GFP. We found that PINK1 MLS-GFP distributed only to the mitochondria and not the cytosol (Figure 2B and 3). This GFP fusion protein was protected from proteinase K digest, suggesting that it is likely localized inside the outer membrane (Figure 2B). As a control, we examined the mito-GFP protein by fractionation, using the cytochrome b2 MLS (1-33 aa). Mito-GFP also resisted proteinase K digest and was not found in the cytosol (Figure 2C). Combined, the data suggests the TM alone is not enough to lead to PINK1 topology with C-terminal portion of the protein facing the cytosol or cytosolic redistribution.

Next we examined our earlier hypothesis that the cleavage after the transmembrane domain allows tethered mitochondrial PINK1 to become cytosolic. Because we are unable to abolish the second PINK1 cleavage with our internal deletion mutants, we constructed and expressed Immt-Δ151 PINK1 fusion protein, one that contains the mitofilin MLS and the PINK1 kinase domain (Figure 1). Mitofilin is a mitochondrial inner membrane protein whose MLS includes a classical presequence followed by a TM, but not a proteolytic site downstream of the TM [24]. We found Immt-Δ151 PINK1 protein localized solely to the mitochondria and its sensitivity to proteinase K suggests an outer membrane topology (Figure 2D and 3). We reasoned that the lack of proteolysis after the TM prevents the release of Immt-Δ151 PINK1 from the mitochondria and it is very likely that Immt-Δ151 PINK1 is tethered to the outer membrane, similar to WT PINK1. The Immt-Δ151 PINK1 construct represents the first successful demonstration that we are able to eliminate the cytosolic pool of PINK1 while retain proper PINK1 mitochondrial topology.

We then asked whether the PINK1 kinase domain itself can confer tethered topology and cytosolic distribution. This time we deleted PINK1 MLS and fused cytochrome b2 MLS to the kinase domain. When we expressed mito-Δ151 PINK1, which now lacks a TM but retains the C-terminal kinase domain, we found this protein distributed equally to the cytosol and the mitochondria. The mitochondrial fraction of mito-Δ151 PINK1 was protected from proteinase K digest, similar to matrix chaperone Hsp60 (Figure 2E and 3). We also examined the subcellular distribution of Δ90-110 PINK1, where the PINK1 TM is deleted. We found that Δ90-110 PINK1 predominantly localized to the mitochondrial fraction that is insensitive to proteinase treatment and a small fraction of cleaved Δ90-110 PINK1 was found in the cytosolic fraction (Figure 2F). Thus in the absence of a transmembrane domain, PINK1 has altered submitochondrial localization but some cytosolic redistribution remains. Taken all together, our data suggests that 1) the TM and the kinase domain are both needed for a tethered, cytosolic-facing, kinase domain topology and 2) PINK1 cytosolic redistribution requires both proteolysis after the TM and the kinase domain.

It was previously shown that PINK1 lacking MLS is mostly cytosolic although it can still interact with OMM or IMS proteins [17,18]. When we expressed Δ151 PINK1, lacking the N-terminal MLS, we found that this protein localized mostly to the cytosol, but some was still found in the mitochondrial fraction and co-localized with mitochondrial markers (Figure 2G and 3). It is likely that Δ151 PINK1 contains additional internal cryptic targeting signal because mitochondrially-localized Δ151 PINK1 was protected from proteinase K digest. Finally, we asked whether or not PINK1 dual distribution is evolutionarily conserved by examining the subcellular localization of drosophila PINK1. We found drosophila PINK1 in both cytosolic and mitochondrial fractions with two cleavage sites similar to the mammalian form (Figure 2H).

**Figure 4:**
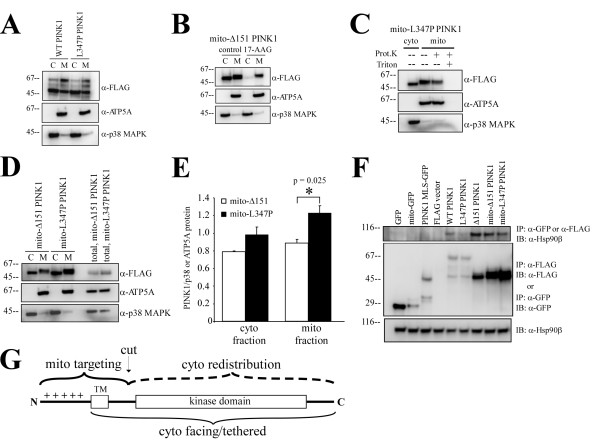
**Hsp90 interaction affects PINK1 subcellular distribution in Hela cells**. A) Subcellular distribution of WT and L347P PINK1 in Hela cells is not noticeably different. C = cytosolic fraction; M = mitochondrial fraction. B) Subcellular distribution of mito-Δ151 PINK1 shows the loss of cytosolic PINK1 when cells are treated with 1 μM 17-AAG, a Hsp90 inhibitor, for 4 hours. C) mito-L347P PINK1 localizes to cytosol and mitochondria. D) Mito-L347P PINK1 accumulates more in the mitochondria than mito-Δ151 PINK1, with little change to the cytosol distribution. E) Quantification from three independent experiments showing more PINK1 in mitochondrial fraction with L347P mutation. P = 0.025. Increase in PINK1 in the cytosolic fraction with L347P is not statistically significant. Mean ± SEM, n = 3. Statistical significance was calculated with ANOVA and Fisher's PLSD post-hoc test. F) L347P mutation reduces PINK1 interaction with Hsp90 by co-immunoprecipitation. GFP does not co-immunoprecipitate with Hsp90. G) Summary diagram depicting PINK1 protein structures and the role of each component in PINK1 topology and subcellular distribution. See Discussion for more details.

To further examine the idea that PINK1 kinase domain-Hsp90 interaction modulates mitochondrial entry of PINK1, we hypothesized that destabilizing the PINK1-Hsp90 interaction will increase PINK1 import into the mitochondria. We wanted to test the idea that the Hsp90 interaction is preventing PINK1 forward movement during mitochondrial import. We chose to use the PINK1 L347P mutation, a naturally occurring PD mutation with reduced Hsp90 interaction [13,25]. First we compared the subcellular localization between PINK1 WT and PINK1 L347P and found there was not observable difference in the cytosolic or mitochondrial distribution between the two proteins (Figure 4A). Even with a loss of Hsp90 binding, we reasoned that the intact transmembrane domain was enough to prevent PINK1 L347P from completely entering the mitochondria. Therefore, we constructed and expressed mito-Δ151 PINK1 where we exchanged the PINK1 MLS with that of cytochrome b2 (1-33aa) to isolate the effect of TM out of the equation and to focus on Hsp90 interaction (Figure 4B). We compared the subcellular distribution of mito-Δ151 PINK1 in the absence and presence of Hsp90 inhibitor, 17-AAG (Figure 4B). We observed that in the presence of 17-AAG, mito-Δ151 PINK1 loses its cytosolic distribution with slight reduction in mitochondrial PINK1. We also noticed that the PINK1 protein sizes are slightly different between cytosol and mitochondria, although we are unsure of the explanation behind this size shift. It has been reported that matrix-localized PINK1 appears as a doublet either through post-translational modification or this size difference may arise from PINK1 having entered the mitochondria to have its MLS cleaved off by mitochondrial matrix protease [19]. In addition to the Hsp90 inhibitor experiment, we constructed mito-L347P PINK1 and compared its subcellular distribution to mito-Δ151 PINK1. When we compared the cytosol/mitochondria distribution between mito-Δ151 PINK1 and mito-L347P PINK1, there was significantly more (p = 0.025) mito-L347P PINK1 (1.226 ± 0.086, mean ± SEM, n = 3) than mito-Δ151 PINK1 (0.888 ± 0.044, mean ± SEM, n = 3) in the mitochondria (Figure 4D-E). Lastly, we confirmed the Hsp90 interaction by co-immunoprecipitation and found a reduction in Hsp90 binding with mito-L347P PINK1 compared to Δ151 or mito-Δ151 PINK1 (Figure 4F). Full length L347P PINK1 also interacted less with Hsp90 compared to WT PINK1, and none of the GFP fusion proteins associated with Hsp90 (Figure 4F). These data suggest that the Hsp90 chaperone interaction on the cytosolic side can prevent PINK1 from further mitochondrial entry, consequentially leading to the release of PINK1 from the mitochondria once proteolysis removes PINK1 from the transmembrane anchor.

## Discussion

As mentioned in the Introduction, both cytosolic and mitochondrial functions of PINK1 have been suggested. Elucidating the exact PINK1 subcellular localization will help us to understand these reported functions. The distribution of PINK1 in cells suggests that while a small percentage of PINK1 can be fully imported or associated with the mitochondria, the majority of PINK1 is believed to reside in the cytosol. The demonstration that PINK1 contains a functional MLS and localizes within the mitochondria supports the hypothesis that PINK1 has a functional role in the mitochondria. While this functional role is unclear, several studies suggest a role of PINK1 in the mitochondrial fission/fusion pathway [26-28] and in mitophagy of damaged mitochondria [22,29-32]. Other compelling scientific data supports the hypothesis that PINK1 is also a cytosolic kinase. Strong evidence of a cytosolic degradation, cytosolic binding partners, and a protective function in the cytosol all point to a kinase protein with a dual localization and possibly two different functions, depending on the subcellular compartment. A major limitation in understanding the subcellular localization of PINK1 is the fact that many studies on PINK1 rely on PINK1 overexpression. Two challenges force researchers to utilize a heterologous overexpression system: the lack of a specific multi-purpose antibody against PINK1 (the anti-PINK1 antibodies are only good for Western blot analysis) and the fact that the endogenous PINK1 expression level is very low. As we have demonstrated previously [12], properties of exogenous PINK1 are reflected by the endogenous PINK1, justifying that overexpressed PINK1 serves as a good model for the endogenous protein.

Unlike other mitochondrial proteins that localize exclusively to the mitochondria, mitochondrial proteins that adopt a cytosolic localization do so in a stimulus-induced fashion. With the exception of yeast fumarase and human PINK1, no other single gene-encoded, MLS-containing protein *constitutively *localizes to both the mitochondria and the cytosol, with the majority of the isoprotein residing in the cytosol. (At least we have not detected others so far.) In this paper, we investigated the important factors for PINK1 topology and dual localization and found three necessary components in the PINK1 protein-the transmembrane domain, the cleavage site after the TM, and the Hsp90 interaction (Figure 4G). We confirmed that the PINK1 MLS is responsible for mitochondrial localization and that two cleavage sites in the PINK1 MLS are responsible for generating PINK1 Δ1 and Δ2, present in both endogenous and exogenous PINK1. We attempted to map out the proteolytic sites by deleting the protein sequence encompassing the predicted cleavage sites. However, PINK1 continued to be cleaved into two products from the precursor. This could mean that we did not target the correct cleavage sites even though they are predicted by MitoPort or other prediction programs. PINK1 presequence cleavage might not follow the classical R-2/R-3/R-10 motif, where there are numerous examples [23,33]. Alternatively, it is thought that cleavage specificity of mitochondrial peptidases is less dependent on the primary protein sequence and more on the structural elements present in both the presequence as well as the mature protein [33]. Thus mutational or deletion studies (as we have done) will have variable results, including a lack of obvious effect on presequence cleavage. What is clear from our internal deletion study is that a second cleavage site is present after the transmembrane domain and this site plays an important role in PINK1 subcellular redistribution. Removal of this second cleavage site completely abolished cytosolic distribution of PINK1, as we showed with a noncleavable TM in mitofilin MLS. Because we are unable to abolish the cleavage of PINK1 MLS, we took advantage of the similarity between PINK1 MLS and mitofilin MLS to determine how presequence cleavage plays a role in PINK1 topology and distribution. Even though Immt-Δ151 PINK1 was not found in cytosol, it was digested by proteinase K, similar to WT PINK1, suggesting that it is tethered to the outer surface of the mitochondria. We predict that if we substitute the PINK1 MLS with a bipartite presequence of an intermembrane space protein (ie cytochrome b2 [3]) then PINK1 would become soluble and redistribute to the cytosol.

When we addressed the role of the transmembrane domain, we confirmed the previous hypothesis that the transmembrane domain, acting as a stop-transfer signal, prevents forward import of PINK1 into the matrix. We demonstrated that in the absence of a transmembrane domain, either by deleting the PINK1 TM or by substituting PINK1 MLS with a matrix targeting signal, we were able to redirect mitochondrial PINK1 into proteinase-insensitive fraction. Thus the transmembrane domain is important, although not sufficient, for membrane tethering and cytosolic-facing topology.

We found that the PINK1 kinase domain, in conjunction with presequence cleavage, contributes to cytosolic redistribution of PINK1. Mitochondrial-targeted GFP (MLS-GFP and PINK1 MLS-GFP) were not found in the cytosol nor was GFP co-immunoprecipitated with Hsp90. When PINK1 kinase domain was present and co-immunoprecipitated with Hsp90, these recombinant proteins all showed dual subcellular distribution, except for IMMT-Δ151 PINK1 (as discussed previously). When we introduced natural PINK1 mutation L347P in the kinase domain, we not only disrupted the Hsp90-PINK1 interaction, we increased the mitochondrial PINK1 level, provided that a TM is absent. More PINK1 L347P mutant protein was found in the mitochondrial fraction compared to its wildtype counterpart. To explain why L347P PINK1 and mito-L347P PINK1 are found in the cytosol, we believe that a complete loss of Hsp90 interaction is necessary, as demonstrated by GFP proteins. In our co-immunoprecipitation experiment, L347P PINK1 and mito-L347P PINK1 showed significant reduction but not a 100% loss of Hsp90 interaction. This residual Hsp90 binding may account for the cytosolic redistribution. Of course, to completely eliminate PINK1-Hsp90 interaction will render PINK1 unstable and destine for rapid proteasome degradation. Importantly, we want to point out that decreased PINK1 retention in the cytosol consists of both accelerated degradation and increased PINK1 mitochondrial entry. When Hsp90 inhibitor, 17-AAG, was used in the experiment for Figure 4B, we did not see an increase in total mitochondrial PINK1 comparing untreated to 17-AAG-we actually saw a loss of signal. This is probably due to accelerated degradation and the loss of total PINK1. Thus we chose to complement the inhibitor data with the L347P mutation experiment-to avoid accelerating PINK1 degradation and other non-specific effects from 17-AAG, thereby to focus on how L347P mutation influences subcellular distribution. In that setting, mitochondrial PINK1 increased. Together, we believe that once PINK1 enters the mitochondria, PINK1 adopts a tethered topology because both the transmembrane domain and the kinase domain prevent PINK1 forward movement into the mitochondria. Subsequent proteolysis downstream of the transmembrane domain then releases PINK1 for retrograde movement while PINK1 kinase interacts with the Hsp90 chaperone.

As demonstrated by Zhou *et al *(2008), we find that PINK1 TM is required for kinase domain facing the cytosol. In addition, PINK1 kinase domain facing the cytosol also requires Hsp90 interaction and we believe it is the combined effects of TM and chaperone interaction that give mitochondrial PINK1 its proper topology. We have demonstrated that PINK1 Δ2 lacks the TM domain and thus its association with mitochondria must be through another mechanism. The question turns to whether or not PINK1 Δ1 is tethered to the mitochondrial membrane? We already know that this PINK1 cleaved form is rapidly degraded by the proteasome. Given the evidence that the first cleavage site might reside within the TM region, this suggests that PINK1 Δ1 might be loosely anchored or not anchored at all in its transient half-life.

## Conclusions

In conclusion, the interaction of the kinase domain with Hsp90 plays a significant role in PINK1 topology and cytosolic redistribution. It is conceivable that Hsp90 binding to the PINK1 kinase domain is preventing the vectorial movement of PINK1 precursor protein during the entire import process. While PINK1 is targeted to the mitochondria, PINK1 function in the mitochondria is unclear. Published results show that loss of PINK1 can lead to mitochondrial dysfunction, but it is not clear that this is the result of losing mitochondrial PINK1 or cytosolic PINK1. Echoing a concern previously raised by Beilina *et al *(2005), the possibility that the cytosol contains mature PINK1 kinase challenges researchers to delineate how exactly PINK1 function links directly to mitochondrial functions. Embedded in this dual subcellular localization model is the proposal that PINK1 has compartment-specific functions, as was found for yeast fumarase. We believe that functional studies of PINK1 need to implement the experimental design of examining PINK1 function when it resides in only one subcellular compartment in order to tease apart PINK1 functional roles.

## Methods

### cDNAs

Mutant *PINK1 *cDNAs were amplified from wildtype human *PINK1 *cDNA via PCR-driven overlap extension [34] with the following primer pairs. The PCR product was then cloned into p3XFLAG-CMV14 (Sigma) flanked by EcoRI and BamHI restriction enzyme sites. Δ25-40 PINK1 (Forward *5' TTC ACG GGC AAG GTC CGC GGA GAG CGT 3';*Reverse *5'ACG CTC TCC GCG GAC CTT GCC CGT GAA 3')*, Δ66-80 PINK1 (Forward *5' CTC GGG CTC CCT AAC TTG CAG CGG CAG TTC 3'; *Reverse *5' GAA CTG CCG CTG CAA GTT AGG GAG CCC GAG 3')*, Δ66-90 PINK1 (Forward *5' CTC GGG CTC CCT AAC GGC TGC GCG GGC CCT T 3'; *Reverse *5' AAG GGC CCG CGC AGC CGT TAG GGA GCC CGA G 3')*, Δ90-110 PINK1 (Forward *5' GTG GTG CGG GCC ATC GAG GAA AAA CAG 3'; *Reverse *5' CTG TTT TTC CTC GAT GGC CCG CAC CAC 3')*, Δ130-150 PINK1 (Forward *5' GTC AGG AGA TCC AGT TTC GGC TGG AGG 3'; *Reverse *5' CCT CCA GCC GAA ACT GGA TCT CCT GAC 3'),*Δ151 PINK1 (Forward *5' ATT GAA TTC AAT GCG GCT GGA GGA GTA TCT G 3'; *Reverse *5' ATA GGA TTA CAG GGC TGC CCT CCA TGA 3')*, L347P PINK1 (Forward *5'CTG CTG CAG CTG CCG GAA GGC GTG GAC 3';R*everse *5' GTC CAC GCC TTC CGG CAG CTG CAG CAG 3')*. Drosophila PINK1 was cloned from Drosophila Gene Collection Clone GH20931 (Open Biosystems) with PCR (Forward *5' CAG AAG CTT ATG TCT GTG AGA CTG CTG AC 3'; Reverse 5' CAG GAT ATC AGC GCC ACC ACA TTC TGG A 3')*

The starting ATG of GFP cDNA in pAcGFP-N1 plasmid (Clontech) was remove by PCR (Forward 5' *CTT GGG ATC CAG TGA GCA AGG GCG CCG A *3'; Reverse 5' *GTC GCG GCC GCT CAC TTG TAC AGC TCA T *3') and GFP was cloned back into the plasmid in BamHI and NotI sites; renamed plasmid GFP-ΔATG. Mito-GFP was cloned by ligating annealed phosphorylated oligos of cytochrome b2 MLS into the EcoRI and BamHI site of GFP-ΔATG (Forward 5' phospho/*AAT TCA TGC TAA AAT ACA AAC CTT TAC TAA AAA TCT CGA AGA ACT GTG AGG CTG CTA TCC TCA GAG CGT CTA AGA CTA GAT TGA ACA CAA TCC GCG CGT ACG GTT CTA CG *3'; Reverse 5' phospho/*GAT CCG TAG AAC CGT ACG CGC GGA TTG TGT TCA ATC TAG TCT TAG ACG CTC TGA GGA TAG CAG CCT CAC AGT TCT TCG AGA TTT TTA GTA AAG GTT TGT ATT TTA GCA TG *3'). To generate PINK1 MLS-GFP, PINK1 MLS was PCR amplified from WT *PINK1 *cDNA and cloned in the EcoRI and BamHI sites of GFP-ΔATG by PCR (Forward 5' *AAG AT TCA ATG GCG GTG CGA CAG GCG 3'*; Reverse *5' ACT GGA TCC CGA AAG CCC TGC AAG C *3'). To generate mito-Δ151 PINK1, Δ151-flag was cloned into EcoRI and NotI of GFP-ΔATG by PCR amplifying Δ151 PINK1 (Forward 5' *ATT GAA TTC CGG CTG GAG GAG TAT CTG *3'; Reverse 5' *ATT GCG GCC GCT CAC TAC TTG TCA TCG TCA T *3'). Then cyt b2 MLS was cloned into XhoI and EcoRI sites by PCR amplifying mito-GFP as template (Forward 5' *ACC CTC GAG ATG CTA AAA TAC AAA CCT TTA C *3'; Reverse 5' *AAA GAA TTC GGT AGA ACC GTA CGC GCG G *3'). To generate Immt-Δ151 PINK1, Immt MLS was PCR amplified and cloned into XhoI and EcoRI sites to replace cyt b2 MLS in mito-Δ151 PINK1 (Forward 5' *AAA CTC GAG ATG CTG CGG GCC TGT C *3'; Reverse 5' *AAT GAA TTC TGA AAG TGC AGG TGT GG *3'). All plasmids were sequence verified. For PINK1 knockdown, siPINK1 oligos (Sense 5' GGA GAU CCA GGC AAU UUU UU 3'; Antisense 5' AAA AAU UGC CUG GAU CUC C 3') were purchased from Applied Biosystems/Ambion. Silencer Negative Control #1 siRNA from Applied Biosystems/Ambion was used as scrambled siRNA control. Oligos were reverse transfected into Hela cells for three consecutive days with siPORT Amine Transfection Agent (Applied Biosystems/Ambion) according to manufacture protocol.

### Cell Culture and Transfection

Hela CCL-2 cells were purchased from ATCC and cultured in DMEM complete media supplemented with 10% FBS and 1% penicillin/streptomycin. Transient transfection method with Lipofectamine 2000 was performed according to manufacturer's protocol (Invitrogen). Briefly, Hela CCL-2 cells were plated onto 60 mm^2 ^tissue culture dishes at 90% confluency at the time of transfection. 2 μg of cDNA was diluted in 250 μL OPTI-MEM. 5 μL of Lipofectamine 2000 was diluted in 250 μL of OPTI-MEM. The mixture of cDNA and Lipofectamine 2000 was added to cells in OPTI-MEM. The transfection media was replaced by DMEM growth media six hours after transfection. Cells were subjected to experiments 48 hours following transfection.

### Co-Immunoprecipitation

Co-IP experiments followed the methods described previously [35]. Briefly, cells were lysed in 1% Triton X-100 buffer (20 mM HEPES [pH 7.5], 150 mM NaCl, 1% Triton X-100, 10% glycerol, 1 mM EDTA, protease inhibitor cocktail, and PMSF). Lysates were rotated for 1 hr at 4°C then cleared by centrifugation at 13,000 g for 10 min at 4°C. Equal protein amount was used for co-IP for all samples. Rabbit anti-FLAG (1:100) or anti-GFP (1:100) antibodies were used for immunoprecipitation at 4°C overnight. 30 μL of Protein A/G PLUS agarose was added the next day, washed three times in 1% Triton X-100 buffer, and resuspended in 2× sample buffer for SDS-HEPES PAGE (Pierce).

### Mitochondrial Isolation

Mitochondria were isolated from Hela CCL-2 cells according to manufacturer's protocol (Pierce) with minor modifications. Briefly, the cells were trypsinized and harvested. A Dounce homogenizer was used to lyse the cells by 70 strokes. After removing the nuclear fraction, the crude supernatant was spun at 3,000 g for 20 minutes to pellet the intact mitochondria. The mitochondrial pellet was resuspended in IP buffer (150 mM NaCl, 50 mM Tris-HCl, 0.5% NP-40, 0.5% sodium deoxycholate, 5 mM EDTA, 0.25% SDS, 0.25 mM PMSF and protease inhibitor cocktail) to collect mitochondrial proteins. For each fractionation, equal amounts of soluble cytosolic protein and mitochondrial protein were determined by BCA assay (Pierce). Proteins were resolved on SDS-HEPES PAGE.

### Proteinase K proteolysis assay

Mitochondria were isolated by the mitochondrial isolation protocol described above. The mitochondrial pellet was resuspended in import buffer (0.6 M sorbitol, 50 mM HEPES, 50 mM KCl, 10 mM MgCl_2_, 2 mM KH_2_PO_4_, pH 7.0 with KOH) and aliquoted into three equal fractions. Final concentration of 50 μg/mL of proteinase K (Qiagen) was added to the appropriate sample tube with or without a final concentration of 1% Triton X-100. Samples were incubated on ice for 30 minutes and the proteolysis was inhibited by the addition of PMSF and protease inhibitor cocktail. Then the samples were centrifuged at max speed for 5 minutes and the pellet was resuspended in IP buffer. Proteins were resolved on SDS-HEPES PAGE.

### Immunocytochemistry

Transfected Hela CCL-2 cells were fixed in paraformaldehyde (4% for 10 min at room temperature) and then washed three times in 0.1% Triton X-100. Antigen retrieval was performed by incubating coverslips in 50 mM Tris-buffered saline, pH 7.5, at 95°C for 20 min, followed by three washes in PBS. Nonspecific immunoreactivity was blocked with 10% goat serum. Cultures were incubated overnight at 4°C in PBS containing a polyclonal FLAG (1:250 dilution) antibody and a monoclonal CoxIV or Hsp90 (1:250 dilution) antibody. Immunoreactivity to FLAG was amplified and detected using an Alexa 488 conjugate of a goat anti-rabbit IgG antibody and CoxIV and Hsp90 were amplified with Alexa 563 conjugate of a goat anti-mouse IgG antibody. The cells were imaged using a 150×, 1.35 NA objective, and optical slices through the cultures were obtained using the 488 and 543 nm lines, respectively, of an Olympus DSU "fixed cell" Spinning Disk Confocal Microscope (Tokyo, Japan) at the Integrated Microscopy Core Facility at the University of Chicago. Images were analyzed with ImageJ (NIH).

### Western blot analysis

Protein quantification was done using the BCA method (Pierce). Immobilon-P PVDF membrane was used in Western blotting (Millipore). After wet transfer, membrane was rinsed briefly with water. The membrane was blocked for 2 hours in blocking buffer (1XTBS containing 5% milk, and 0.1% tween-20). Appropriate primary antibodies were incubated for overnight in blocking buffer, and secondary antibodies were incubated in room temperature for 1 hour in blocking buffer. The membrane was then developed with ECL reagents (Milipore) and imaged with ChemiGenius Bio-Imaging system (Syngene). Optical density of protein signals were measured with ImageJ. Endogenous PINK1 was detected using Odyssey Infrared Imaging System (LI-COR Biosciences).

### Antibodies and Chemicals

The following antibodies were purchased commercially. Anti-PINK1 (BC100-494) 1:1000 (Novus Biological), anti-FLAG 1:100 (Sigma), anti-FLAG M2 1:5000 (Sigma), anti-Hsp90β 1:250 (Santa Cruz), anti-β actin 1:20,000 (Sigma), anti-p38 MAPK 1:2000 (Cell Signal Technologies), anti-CoxIV 1:200 (Invitrogen), anti-mouse IgG-HRP 1:20,000 (Promega), anti-goat IgG-HRP 1:1000 (Promega), anti-rabbit IgG HRP 1:10,000 (Promega), anti-ATP5A 1:10,000 (BD Biosciences), anti-Hsp60 1:10,000 (Cell Signal Technologies), anti-GFP 1:10,000 (Clontech). All chemicals are from Sigma, unless noted.

### Statistical Analysis

Statistics were calculated with ANOVA followed by Fisher's PLSD post-hoc test for significance at 5% with Statview software (SAS Institute, NC, USA).

## Abbreviations

PINK1: PTEN-induced putative kinase 1; OMM: Outer mitochondrial membrane; MLS: Mitochondrial localization signal; GFP: Green fluorescent protein; FL: Full-length; TM: transmembrane; AA: amino acid;

## Authors' contributions

WL co-designed the study, carried out all the experiments, performed data analysis, and co-wrote the manuscript. UJK co-designed the study, analyzed the data, and co-wrote the manuscript. Both authors read and approved the final manuscript.
